# Living functional hydrogels generated by bioorthogonal cross-linking reactions of azide-modified cells with alkyne-modified polymers

**DOI:** 10.1038/s41467-018-04699-3

**Published:** 2018-06-06

**Authors:** Koji Nagahama, Yuuka Kimura, Ayaka Takemoto

**Affiliations:** Department of Nanobiochemistry, Frontiers of Innovative Research in Science and Technology (FIRST), Konan University, 7-1-20 Minatojima-Minamimachi, Chuo-ku, Kobe 650-0047 Japan

## Abstract

To date, many scientists have thoroughly investigated both cells and cellular functions, resulting in the identification of numerous molecular mechanisms underlying the cellular functions. Based on these findings, medical scientists and pharmacologists have developed many technological applications for cells and cellular functions in medicine. How can material scientists utilize cells and cellular functions? Here, we show a concept for utilizing cells and their functions from the viewpoint of materials science. In particular, we develop cell cross-linked living bulk hydrogels by bioorthogonal click cross-linking reactions of azide-modified mammalian cells with alkyne-modified biocompatible polymers. Importantly, we demonstrate the unique functionalities of the living hydrogels, originating from the basic functions of the cells incorporated in the living hydrogels as active cross-linking points. The findings of this study provide a promising route to generating living cell-based next-generation innovative materials, technologies, and medicines.

## Abstract (web summary)

Cells are often passively encapsulated in hydrogels for a wide range of applications. Here, the authors report on a method of incorporating the cells into the hydrogel using bioorthogonal click reactions to link azide-modified cells with biocompatible polymers to create cell-based materials.

## Introduction

Many scientists have been and continue to be interested in cells, and especially in cellular functions. This has led to the identification of many molecular mechanisms underlying cellular functions and cell–cell interactions in living systems^1–5^, which in turn has led to the development by medical scientists and pharmacologists of many technological applications of cells and cellular functions in medicine, including cancer therapy and regenerative medicine^6–8^. How can materials scientists utilize cells and cellular functions? The molecular mechanisms underlying cellular functions provide the best role models for the design of advanced multifunctional materials, and chemists have utilized functional biomolecules, such as nucleic acids^9, 10^, proteins^11, 12^, and polysaccharides^13, 14^, as essential active components for designing materials, including smart materials. Cells and cellular functions are also attractive and promising active components for the design of functional materials. Combining living cells with synthetic materials could enable the fabrication of living multifunctional materials capable of, for example, sensing the environment, time-programming, movement, and signal transduction, all originating from the functions of the incorporated cells.

**Fig. 1:**
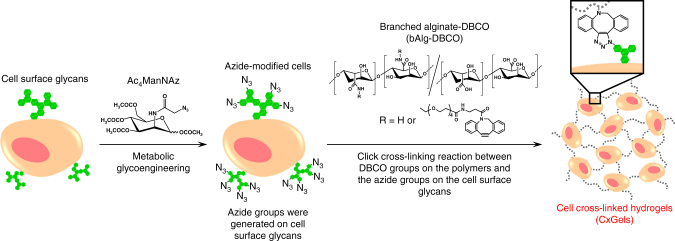
Schematic illustration of the construction of cell cross-linked hydrogels (CxGels). Reactive azide groups are covalently incorporated into cell-surface glycans through the biosynthetic machinery. CxGels are constructed via bioorthogonal click cross-linking reaction between the azide-modified cells and the alkyne-modified polymers

Here, we demonstrate a concept for utilizing cells and their functions from the viewpoint of materials science. Specifically, we demonstrate living multifunctional hydrogels generated by bioorthogonal click cross-linking reactions of azide-modified mammalian cells with alkyne-modified biocompatible polymers, as shown in Fig. 1. Furthermore, we demonstrate the unique functionality of the living hydrogels originating from the basic functions of the incorporated cells as active cross-linking points.

## Results

### Preparation of cell cross-linked hydrogels

Metabolic glycoengineering was used to incorporate reactive azide groups on the cell surface^15–17^. The monosaccharide precursor was modified with an azide group, then incorporated into cell-surface glycans through biosynthetic machinery. Sialic acid is one of the most abundant cell-surface glycans on mammalian cells and is typically found at the terminating branches of these glycans^18, 19^. We therefore targeted sialic acid residues for azide-modification because the location (the outermost surface of cells) and abundance (high concentration on cell surface) of sialic acid residues is ideal for efficient bioorthogonal click cross-linking with alkyne-modified polymers. The tetraacetylated monosaccharide *N*-azidoacetylmannosamine (AC_4_ManNAz) was synthesized as the precursor for azide-modified sialic acid residues, as reported previously (Supplementary Fig. 1)^20, 21^. The obtained AC_4_ManNAz was characterized by ESI-MS and ^1^H-NMR measurements (Supplementary Figs. 2 and 3). Conversion of the NH_2_ group of mannosamine into an azide groups was calculated to be 96% based on the ^1^H-NMR spectrum and conversion of the OH groups of *N*-azidomannosamine into acetyl groups was estimated to be 97%. AC_4_ManNAz was not cytotoxic to C2C12 cells (mouse myoblast) below 100 μM (Supplementary Fig. 4). Following treatment with AC_4_ManNAz, azide groups on the cell surface were detected by covalent labeling using the clickable fluorescent dye dibenzocyclooctyne (DBCO)-modified carboxyrhodamine. Fluorescence microscopic images (Supplementary Fig. 5a) showed surface-labeled C2C12 cells, indicating the incorporation of azide groups on the cell surface glycans. The fluorescence intensity per cell was quantified and clearly increased as the AC_4_ManNAz concentration increased (Supplementary Fig. 5b). Moreover, growth curves of azide-modified C2C12 cells [N_3_(+)C2C12] treated with 100 μM AC_4_ManNAz were similar to that of normal C2C12 cells [N_3_(−)C2C12] (Supplementary Fig. 6). We therefore chose 100 μM as the optimal AC_4_ManNAz concentration. Importantly, cell-surface fluorescence was maintained even after 10 days’ cultivation in DMEM without AC_4_ManNAz, although the fluorescence intensity gradually decreased due to cell division (Supplementary Fig. 7).

Live cells were covalently cross-linked with alkyne-modified polymers using copper-free click chemistry to avoid the potential toxicity of copper catalysts. We selected alginic acid (Alg, 100,000 Da) as a polymer component because of its good biocompatibility and synthesized branched alginic acid (bAlg) using amine-terminated 4-arm branched polyethylene glycol (4-arm PEG, 20,000 Da, Supplementary Fig. 8). The molecular composition of bAlg was estimated by ^1^H-NMR analysis (Supplementary Fig. 9). The integration ratios of the anomeric protons of glucuronic acid (peak a) and mannuronic acid (peak b) in the Alg segment to the methylene protons of the PEG segment (peak c) indicated that the molar ratio of Alg to 4-arm PEG in bAlg was ~9:1. Next, we synthesized dibenzocyclooctyne (DBCO)-modified bAlg (bAlg-DBCO) (Supplementary Fig. 10); ^1^H-NMR analysis showed that on average 13 DBCO groups were introduced per bAlg molecule and the molecular weight of bAlg-DBCO was 1,026,800 Da (Supplementary Fig. 11). We analyzed the hydrodynamic diameter of bAlg-DBCO by dynamic light scattering (DLS) using a dilute solution (0.05%) of bAlg-DBCO and found that the hydrodynamic diameter was approximately 0.8 μm (Supplementary Fig. 12).

**Fig. 2:**
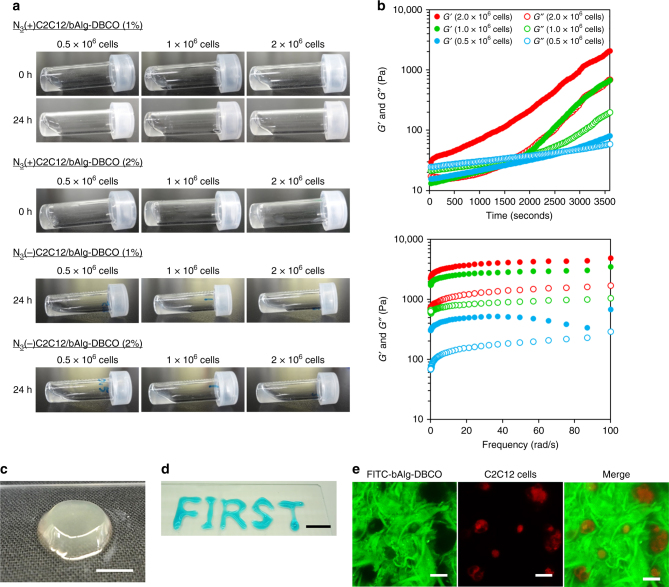
Preparation and characterization of cell cross-linked hydrogels (CxGels). **a** Photographs of reaction mixtures of azide-modified C2C12 cells (0.5 × 10^6^, 1.0 × 10^6^, and 2.0 × 10^6^ cells) and bAlg-DBCO solutions (1% and 2%, w/v) at 0 h and 24 h after the reaction. Photographs of dispersions of C2C12 cells (0.5 × 10^6^, 1.0 × 10^6^, and 2.0 × 10^6^ cells) and bAlg-DBCO solutions (1% and 2%, w/v) after 24 h. **b** (upper) Gelation kinetics determined through oscillatory time sweep of CxGels prepared from bAlg-DBCO (2%) and azide-modified C2C12 (0.5 × 10^6^, 1.0 × 10^6^, and 2.0 × 10^6^) at 37 °C under constant strain (5%) and frequency (10 rad/s). The crossover time point of the storage modulus (*G*′) and the loss modulus (*G*″) curves is defined as the mechanical gel point. (lower) Frequency sweep of CxGels prepared from bAlg-DBCO (2%) and azide-modified C2C12 (0.5 × 10^6^, 1.0 × 10^6^, and 2.0 × 10^6^) at 2 h after the start of the cross-link reactions at 37 °C under constant strain (5%). **c** Photographs of CxGels prepared through click reaction between azide-modified C2C12 cells (2.0 × 10^6^ cells) and bAlg-DBCO solution (2%). Scale bar indicates 5 mm. **d** Photographs of the word FIRST written in CxGel made through click reaction between azide-modified C2C12 cells (2.0 × 10^6^ cells) and bAlg-DBCO solution (2%). CxGels were stained with Fast Green FCF to aid visualization. Scale bar indicates 10 mm. **e** CLSM images of CxGels made through click reaction between azide-modified C2C12 cells (2.0 × 10^6^ cells) and bAlg-DBCO solution (2%). Green: FITC-bAlg-DBCO, red: azide-modified C2C12 cells stained with CytoTell Red. Scale bars indicate 20 μm

Next, we investigated the click reaction between N_3_(+)C2C12 cells and bAlg-DBCO by suspending N_3_(+)C2C12 cells in PBS and reacting with FITC-labeled bAlg-DBCO at 37 °C for 30 min. Fluorescence microscopic images (Supplementary Fig. 13) showed C2C12 cells with green fluorescence on their surface, demonstrating the successful bioorthogonal click reaction between bAlg-DBCO and the azide groups on the cell surface glycans. The number of N_3_(+)cells and the concentration of bAlg-DBCO can be critical factors for the construction of cell cross-linked hydrogels and thus cell cross-linking reactions were performed with different numbers of N_3_(+)cells and different bAlg-DBCO concentrations. Hydrogel formation was analyzed using the classic test tube inversion method and by rheological characterization. First, oscillatory time sweep and frequency sweep analyses of 2% bAlg-DBCO solutions showed that the *G*″ values were always larger than the *G*′ values, indicating that the bAlg-DBCO solution was in a sol state (Supplementary Fig. 14a). We performed a gelation study using a combination of N_3_(−)C2C12 cells and bAlg-DBCO solutions, using combinations of N_3_(+)C2C12 cells and bAlg solutions as negative controls. As shown in Fig. 2a and Supplementary Fig. 14, we confirmed that these negative controls did not result in hydrogel formation. Reaction mixtures of N_3_(+)C2C12 cells and bAlg-DBCO solution (1%) did not form bulk-sized hydrogels even after 24 h (Fig. 2a). In contrast, immediate gelation was achieved using a reaction mixture of 2.0 × 10^6^ N_3_(+)C2C12 cells and 2% bAlg-DBCO solution (Fig. 2a and Supplementary Movie 1).

### Characterizations of cell cross-linked hydrogels

We investigated the gelation time by performing oscillatory time sweep measurements of reaction mixtures comprising various numbers of N_3_(+)C2C12 cells (0.5 × 10^6^, 1.0 × 10^6^, 2.0 × 10^6^) and a fixed amount of 2% bAlg-DBCO solution (Fig. 2b). Importantly, the *G*′ value of a mixture of 2.0 × 10^6^ N_3_(+)C2C12 cells and 2% bAlg-DBCO solution was already higher than their *G*″ value at time zero (just after mixing by pipetting), proving that cell cross-linked hydrogels (CxGels) are formed via bioorthogonal click reactions between bAlg-DBCO and N_3_( + )C2C12 cells. The gelation time of the CxGels decreased as the number of N_3_(+)C2C12 cells increased, indicating that the N_3_( + )C2C12 cells are a critical factor in facilitating the fabrication of networks of bAlg polymers. Frequency sweep experiments were performed on the CxGels were performed 2 h after initiation of the cross-linking reaction. The *G*′ values of CxGels containing relatively high cell numbers (1.0 × 10^6^ and 2.0 × 10^6^) did not change with angular frequency, suggesting that the hydrogels were stably formed. In contrast, the *G*′ values of CxGels containing a low cell number (0.5 × 10^6^) changed at high frequency, suggesting that the bAlg networks in the hydrogels were defective. The *G*′ values of the CxGels increased as the number of N_3_(+)C2C12 cells increased, indicating that the mechanical strength of the CxGels was governed by the number of N_3_(+)C2C12 cells. These results strongly indicate that N_3_(+)C2C12 cells act as cross-linking points for the fabrication of three-dimensional bAlg networks in CxGels, as illustrated in Fig. 1. Interestingly, CxGels remained free-standing even after vortex shaking (Fig. 2c). Moreover, CxGels prepared using 2.0 × 10^6^ N_3_(+)C2C12 cells and 2% bAlg-DBCO solution can be molded immediately into complex shapes by discharging the reaction mixture from a pipet (Fig. 2d), indicating good handling and molding properties. Furthermore, CxGels can be constructed using several kinds of cells (Supplementary Fig. 15a and 12b), indicating that this gel construction method is universally applicable to whole mammalian cells. Note that, since N_3_(+)C2C12 cells can be stored in the frozen state for long periods of time and retain their azide-modification, CxGels can be made using thawed cells (Supplementary Fig. 15c). Long-term storage is advantageous for securing a stable supply of N_3_(+)cells for use as living building blocks. Confocal microscopy analysis of CxGels revealed that C2C12 cells (labeled with red) were uniformly dispersed within the three-dimensional networks of bAlg (labeled with green) (Supplementary Fig. 16). Moreover, microphase separated structures consisting of cell domains and bAlg networks were observed (Fig. 2e), indicating that bAlg did not enter the cells but rather reacted with the surface of the cells. We calculated the total volume fraction of C2C12 cells in a CxGel prepared by mixing 2.0 × 10^6^ N_3_(+)C2C12 cells and 100 μL of 2% bAlg-DBCO solution by using a simple sphere model for the cells and assuming that the radius of each cell is 10 μm. The volume of a cell was calculated to be 4187 μm^3^ using the formula (4/3)pi*r^3^. The total volume of cells in the CxGel was 8.4 × 10^9^ μm^3^, and thus the total volume fraction of C2C12 cells in the CxGel was 8.4%. The total volume fraction of C2C12 cells likely corresponds to the three-dimensional image of a CxGel obtained by confocal microscopy analysis (Supplementary Fig. 16).

**Fig. 3:**
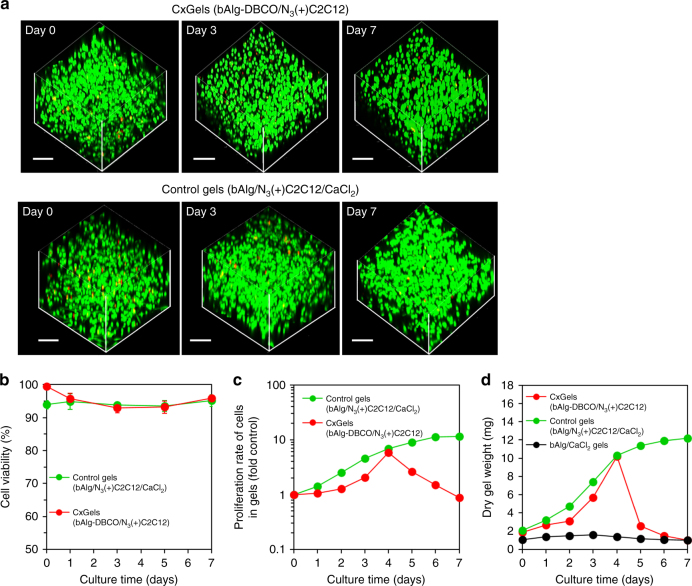
Characterizations of the CxGels. **a** (upper) CLSM images of CxGels prepared through click reaction between azide-modified C2C12 cells (2.0 × 10^6^ cells) and bAlg-DBCO solution (2%) after live/dead assay. (lower) CLSM images of C2C12 cells-encapsulating control physical gels prepared through physical cross-linking reaction between bAlg solution (2%) and CaCl_2_ solution (0.5%) in the presence of azide-modified C2C12 cells (2.0 × 10^6^ cells) after live/dead assay. CLSM observation was carried out at days 0, 3 and 7 after CxGels preparation. Green fluorescence indicates live cells stained with calcein-AM and red fluorescence indicates dead cells stained with PI. Scale bars indicate 200 μm. **b** Cell viability of C2C12 cells in the CxGels prepared through click reaction between azide-modified C2C12 cells (2.0 × 10^6^ cells) and bAlg-DBCO solution (2%) and the cell-encapsulating control physical gels analyzed using the CLSM images after live/dead assay. Live/dead assay was performed at days 0, 1, 3, 5, and 7. Error bars: standard deviation (*n* = 3). **c** Proliferation rates of C2C12 cells existing in the CxGels prepared through click reaction between azide-modified C2C12 cells (2.0 × 10^6^ cells) and bAlg-DBCO solution (2%) and the control physical gels analyzed using the WST-1 assay. Error bars: standard deviation (*n* = 3). **d** Time-dependent changes in the dry CxGels weight, the cell-encapsulating control physical gel, and normal bAlg physical gels cultured in DMEM for 7 days at 37 °C. Error bars: standard deviation (*n* = 3)

A key question regarded the fate of cells in CxGels acting as active cross-linking points was: do the cells remain alive and grow in the CxGels? The viability of C2C12 cells in CxGels was investigated using a live/dead assay. As shown in Fig. 3a, b, most of the C2C12 cells in the CxGels showed green fluorescence, with only a few cells showing red fluorescence, indicating high cell viability (over 93%). Next, we investigated the proliferation of C2C12 cells in CxGels using the WST-1 assay and obtained a linear relationship between the cell number and the absorbance within the cell concentration range tested (Supplementary Fig. 17). The C2C12 cells in CxGels clearly proliferated by logarithmic growth, suggesting that cells in CxGels exhibit basic cellular functions. As mentioned above, our aim in this study is to demonstrate that the covalent combination of living cells, acting as active cross-linking points, enables the development of multifunctional hydrogels with unique functionalities that originate from the cells. We therefore selected two basic cellular functions to demonstrate utility of our approach: autonomous cell growth and selective cell adhesion.

### Self-growing and self-degradation abilities of cell cross-linked hydrogels

First, autonomous cell growth in CxGels was used to endow functionality to the CxGels. We investigated time-dependent changes in the swelling ratio of the CxGels, the number of C2C12 cells in the CxGels, their dry gel weight, and the mechanical strength of the gels during 7 days’ cultivation. Furthermore, we compared these physical properties with those of appropriate control gel: N_3_(+)C2C12 cells encapsulated in bAlg gels physically cross-linked with calcium ions (gels in which the cells are not cross-linked with bAlg) and bAlg/Ca^2+^ physical gels (gels which do not contain cells). No significant change in the swelling ratio or dry weight of the bAlg/Ca^2+^ gels were observed during the 7 days (Fig. 3d; Supplementary Fig. 18), indicating that the bAlg/Ca^2+^ gels were not degraded under these experimental condition. Note that the CxGels showed remarkably higher swelling ratios than the C2C12 cell-encapsulating bAlg/Ca^2+^ gels. Alginate is inherently non-degradable in cell culture medium, as medium lack enzymes that can cleave alginate polymer chains. In contrast, ionically physically cross-linked alginate gels can be swollen and then eroded by the release of Ca^2+^ ions into the surrounding medium due to exchange reactions with monovalent cations such as sodium ions^22^. Consequently, the swelling of C2C12 cell-encapsulating bAlg/Ca^2+^ gels would be due to exchange reactions with monovalent cations produced by cell metabolism processes. On the other hand, the CxGels in which the bAlg network is covalently cross-linked with cells should be resistant to swelling caused by ion exchange reaction. However, since cells act as the cross-linking points in the CxGels, cell division in the CxGels would decrease the number of cross-linking points, leading to swelling.

The dry weights of the CxGels and the C2C12 cell-encapsulating bAlg/Ca^2+^ gels increased in a time-dependent manner over the first 4 days and the rate of increase of the C2C12 cell-encapsulating bAlg/Ca^2+^ gels was higher than that of the CxGels (Fig. 3d). The number of C2C12 cells in both the CxGels and bAlg/Ca^2+^ gels increased in a time-dependent manner but the proliferation rate in the bAlg/Ca^2+^ gels was higher than that in the CxGels (Fig. 3c). These results suggest that the increase in dry weight of both the CxGels and the C2C12 cell-encapsulating bAlg/Ca^2+^ gels is due to cell proliferation in these gels. To verify this interpretation, we examined the relationship between the number of C2C12 cells and their dry weights: we obtained a linear relationship, and the dry weight per 1.0 × 10^6^ cells was estimated to be 0.9 mg. This incremental increase in dry weights per 1.0 × 10^6^ cells roughly corresponds with the dry weight of C2C12 cells initially present in these gels, although the dry weight of the gel would include the cell culture medium, metabolites, and ECM proteins produced by the cells. Note that the dry weight of the CxGels decreased from day 4 to day 7, and the number of C2C12 cells present in the CxGels also decreased during this time (Fig. 3d). On the other hand, the dry weight of the C2C12 cell-encapsulating bAlg/Ca^2+^ gels continuously increased during this 7 day periods, and the number of C2C12 cells in the bAlg/Ca^2+^ gel also increased during this time. Changes in the mechanical strength of the CxGels during cultivation are shown in Supplementary Fig. 19. The *G*′ values of the CxGels decreased in a time-dependent manner, and remarkable changes in the *G*′ values at high frequency were detected for CxGels cultured for 5 and 7 days, suggesting that the bAlg networks in these hydrogels were defective. Taking all our results together, we conclude that since C2C12 cells act as the cross-linking points in CxGels, the cell proliferation (cell division) causes a decrease in the number of cross-linking points, leading to swelling (from day 1 to day 4) and subsequent degradation (from day 4 to day 7) of the CxGels, as illustrated in Supplementary Fig. 20. In other words, components of CxGels are released after day 4, leading to a decrease in dry gel weight. Thus, in the CxGel system, cell proliferation (cell division) directly affects the swelling and degradation properties of the CxGels but does not directly affect the swelling and degradation properties of C2C12 cell-encapsulating bAlg/Ca^2+^ gels. Therefore, CxGels have the ability to self-grow and self-degrade due to the autonomous growth of cells utilized as active cross-linking points, and we propose that these are unique properties of CxGels.

### Selective adhesion ability of cell cross-linked hydrogels

**Fig. 4:**
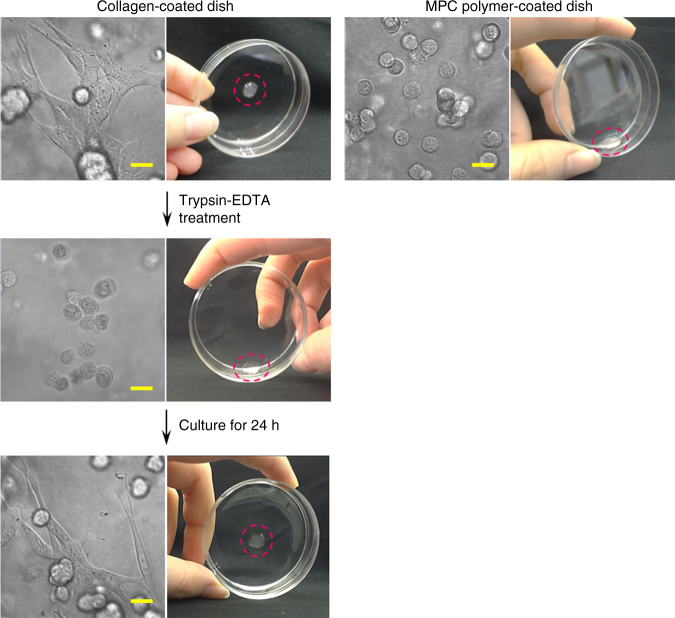
CxGel adhesion to cell culture dishes via the adhesion of cells at the surface. (left panels) Photographs of CxGels, prepared through click reaction between azide-modified C2C12 cells (2.0 × 10^6^ cells) and bAlg-DBCO solution (2%), adhered to collagen-coated dishes via the adhesion of cells in the CxGels. After trypsin-EDTA treatment, the adhering cells detached from the dish and the CxGels detached. The CxGels adhered onto a collagen-coated dish again when placed on the dish and cultured in DMEM for 24 h. (right panel) In contrast, since cells in the CxGels, prepared through click reaction between azide-modified C2C12 cells (2.0 × 10^6^ cells) and bAlg-DBCO solution (2%), did not adhere onto MPC polymer-coated dishes, the CxGel also did not adhere onto MPC polymer-coated dishes even after 24 h. Scale bars indicate 20 μm

Second, we utilized the selective adhesion properties of cells. We performed adhesion studies of CxGels and of the C2C12 cells in CxGels, together with two appropriate control gels: N_3_(+)C2C12 cell-encapsulating bAlg/Ca^2+^ gels and bAlg/Ca^2+^ gels. The gels were placed on collagen-coated dishes or 2-methacryloyloxyethyl phosphorylcholine (MPC) polymer-coated dishes and cultured for 24 h in DMEM. An MPC polymer coating resists the surface binding of cells^23^. We found that the bAlg/Ca^2+^ physical gels did not adhere to either the collagen- or MPC polymer-coated dishes (Supplementary Fig. 21), indicating that no effective interaction is formed between bAlg molecules and collagen- or MPC polymer-coated dishes. Moreover, the bAlg/Ca^2+^ gels encapsulating C2C12 cells also did not adhere onto either dish, whereas C2C12 cells physically encapsulated in bAlg/Ca^2+^ gels adhered onto the collagen-coated dishes. This result indicates that the adhesion of C2C12 cells physically encapsulated in networks of bAlg/Ca^2+^ gels did not result in the adhesion of the entire gel. On the other hand, C2C12 cells in CxGels cultured on a MPC polymer-coated dish did not adhere and the CxGels slipped on the dish when tilted (Fig. 4 right panel and Supplementary Movie 2). In contrast, C2C12 cells in CxGels cultured on a collagen-coated dish adhered and the CxGels also adhered to the dish (Fig. 4 top of left panel). Consequently, the adhesion of C2C12 cells chemically (covalently) connected to the bAlg networks of CxGels results in adhesion of the entire gel. Surprisingly, the CxGels maintained adhesion even after relatively strong physical stimuli (Supplementary Movie 3 and 4). However, trypsin-EDTA treatment resulted in the adhering CxGels easily detaching from the dish due to detachment of the adhering cells from the dish (Fig. 4, middle of left panel and Supplementary Movie 5), indicating that detachment of the cells directly results in detachment of the CxGels. Thus, the selective adhesion of CxGels is derived from the ability of C2C12 cells in CxGels to selectively adhere onto surfaces. Interestingly, the detached CxGels can adhere again onto a collagen-coated dish after 24 h’ cultivation in DMEM due to the reversible adhesion of cells in the CxGels (Fig. 4 bottom left panel and Supplementary Movie 6).

### Preparation of tissue cross-linked hydrogels

**Fig. 5:**
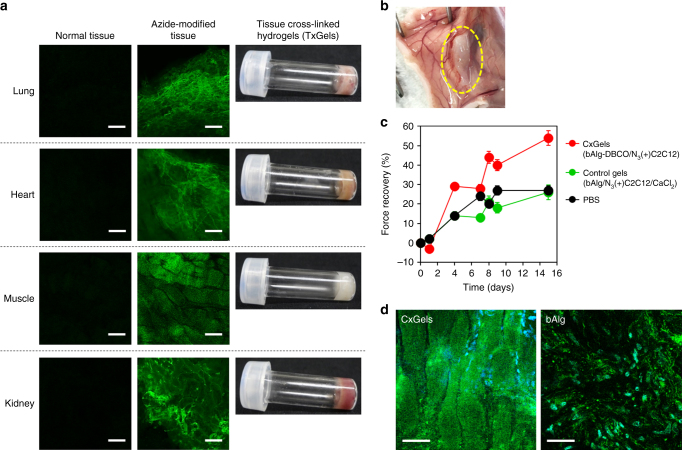
Tissue cross-linked hydrogels (TxGels) and in vivo CxGels formation. **a** (left panels) CLSM images of normal mouse tissues treated with FITC-bAlg-DBCO solution. These tissues were carefully excised, then shredded and suspended with 500 µL of FITC-labeled bAlg-DBCO (0.1%) solution at 37 °C for 30 min. After that, tissues were centrifuged and washed with PBS twice, then tissues were re-suspended with 1 mL of Live Cell Imaging Solution and observed by CLSM. Scale bars indicate 100 μm. (middle panels) CLSM images of azide-modified mouse tissues treated with FITC-bAlg-DBCO solution. Mice treated with AC_4_ManNAz for continuous 7 days were killed and the tissues were excised carefully. These tissues were shredded and washed with PBS twice. These tissues were carefully excised, then shredded and suspended with 500 µL of FITC-labeled bAlg-DBCO (0.1%) solution at 37 °C for 30 min. After that, tissues were centrifuged and washed with PBS twice, then tissues were re-suspended with 1 mL of Live Cell Imaging Solution and observed by CLSM. Scale bars indicate 100 μm. (right panels) Photographs of tissue cross-linked hydrogels (TxGels) made through click reaction between azide-modified tissues and bAlg-DBCO solution (2%) ex vivo. Scale bars indicate 100 μm. **b** Photographs of in vivo formed CxGels. LifeAct-GFP-expressing azide-modified C2C12 cells (4.0 × 10^6^) were suspended with 200 μL of bAlg-DBCO solution (2%) and immediately injected subcutaneously into the muscle layer of the back of each mouse. **c** Force recovery of injured femoral muscle treated with CxGels prepared through click reaction between LifeAct-GFP-expressing azide-modified C2C12 cells (5.0 × 10^6^) and 250 μL of bAlg-DBCO (2%) or C2C12 cells-encapsulating control physical gels, prepared through physical cross-linking reaction between 250 μL of bAlg solution (2%) and CaCl_2_ solution (0.5%) in the presence of azide-modified C2C12 cells (5.0 × 10^6^ cells). Error bars: standard deviation (*n* = 3). **d** Fluorescence images of LifeAct-GFP-expressing C2C12 cells in the CxGels or the control physical gels acquired 15 days after transplantation into injured muscle. Green: LifeAct-GFP, blue: Hoechst. Scale bars indicate 50 μm

To assess whether our approach to fabricate cell cross-linked hydrogels is applicable to cells in tissues, we attempted the in vivo azide-modification of cells in tissues via the intraperitoneal administration of Ac_4_ManNAz to mice for 7 continuous days (Supplementary Fig. 22). The azide-modification of lung, femoral muscle, kidney, and heart tissues was assessed by excising the tissue and treating with FITC-labeled bAlg-DBCO ex vivo. Control tissues (without Ac_4_ManNAz administration) did not show green fluorescence, whereas tissues from mice subjected to Ac_4_ManNAz administration clearly showed green fluorescence (Fig. 5a left and middle panel), indicating the successful in vivo reactive azide-modification of tissues. We prepared tissue cross-linked hydrogels (TxGels) via a bioorthogonal click cross-linking reaction between azide-modified tissues and bAlg-DBCO. Interestingly, all tissues tested formed TxGels, indicating that our approach for fabrication of CxGels is applicable to cells in tissues (Fig. 5a right panel).

### Potential application of cell cross-linked hydrogels as injectable biomaterials for regenerative medicine

We conceived the design of injectable gels for regenerative medicine as one example of the potential application of CxGels and thus investigated in vivo CxGel formation. A suspension of LifeAct-GFP-expressing N_3_(+)C2C12 cells in bAlg-DBCO solution was injected into the subcutaneous tissue of nude mice, and in situ hydrogel formation was observed (Fig. 5b). To assess the utility of CxGels in regenerative medicine, LifeAct-GFP-expressing N_3_(+)C2C12 cells suspended in bAlg-DBCO solution were injected into severe skeletal muscle defects in the hind limbs of mice (Supplementary Fig. 23) and functional recovery was evaluated using a grip-strength meter^24^. We also performed this muscle injury study using two appropriate negative controls: treatment with bAlg/Ca^2+^ physical gels encapsulating LifeAct-GFP-expressing N_3_(+)C2C12 cells, and no treatment (PBS injection). Note that the CxGels formed in situ clung to the muscle defect site due to the selective adhesion ability of the C2C12 cells in CxGels. Mice transplanted with CxGels exhibited remarkably higher force recovery as compared with mice treated with bAlg/Ca^2+^ gels encapsulating C2C12 cells (Fig. 5c) while mice treated with the control gels exhibited similar force recovery as untreated mice. Importantly, GFP-expressing muscle tissue-like structures were detected within the CxGels at day 15 after transplantation (Fig. 5d) whereas no organized structures were observed in mice treated with the control gels. We therefore found that treatment with CxGels provides the most effective muscle force recovery, demonstrating the advantage of CxGels. Moreover, these results indicate the utility of CxGels as delivery and scaffolding materials for regenerative medicine.

## Discussion

Cells and cellular functions should be attractive and promising active components for the design of functional materials. Combining living cells with synthetic materials could enable the fabrication of living multifunctional materials capable of, for example, sensing the environment, time-programming, movement, and signal transduction, all originating from the functions of the incorporated cells. In this study, we present a concept for utilizing cells and their functions from the viewpoint of materials science. In particular, we develop cell cross-linked living bulk hydrogels by bioorthogonal click cross-linking reactions of azide-modified mammalian cells with alkyne-modified biocompatible polymers (bAlg-DBCO). As mentioned above, our aim in this study is to demonstrate that the covalent combination of living cells, acting as active cross-linking points, enables the development of multifunctional hydrogels with unique functionalities that originate from the cells. We therefore selected two basic cellular functions to demonstrate utility of our approach: autonomous cell growth and selective cell adhesion. First, we found that the cell proliferation (cell division) directly affects the swelling and degradation properties of the CxGels. Therefore, CxGels have the ability to self-grow and self-degrade due to the autonomous growth of cells utilized as active cross-linking points, and we successfully demonstrate that these are unique properties of CxGels. Second, we found that the selective adhesion of CxGels is derived from the ability of cells in CxGels to selectively adhere onto surfaces. Hydrogels generally have a remarkably low friction coefficient because of the large amount of free water on their surface, making the stable attachment of hydrogels onto solid materials difficult^25^. On the other hand, cells can adhere onto various solid materials with a wide range of water contact angles in the presence of cell attachment proteins^26^. We found that CxGels can adhere onto materials which alginate gels cannot by using the selective adhesion abilities of the cells. Thus, we propose that the selective adhesive ability of CxGels is unique as compared to existing hydrogels. Taken together, we have successfully demonstrated that the functions of cells covalently incorporated into CxGels as active cross-linking points are useful for endowing CxGels with unique functionalities.

In this study, we prepared tissue cross-linked hydrogels (TxGels) via a bioorthogonal click cross-linking reaction between azide-modified tissues and bAlg-DBCO. Recently, organ on a chip technology has received significant attention as an exciting approach to test chemicals for human safety^27, 28^. In this technology, three-dimensional cellular assemblies with organ-level structures and functions must be re-created on a chip. The success of organ on a chip technology requires the development of methods to create three-dimensional cellular assemblies applicable to all types of cells in an organism. In this context, we conceived that the fabrication of CxGels would be applicable to creating three-dimensional cellular assemblies of various types of cells. Generally, tissues exhibit more complicated, diverse, and higher functionalities than single cells, and thus TxGels likely exhibit unique, higher order functionalities than CxGels. This approach to the fabrication of TxGels would be applicable to organ on a chip technology.

In conclusion, we demonstrated a concept and a method for utilizing cells and cellular functions in the design of multifunctional bulk hydrogels. Importantly, whole mammalian cells and their functions are retained in CxGels, and unique functionalities are generated. This method can be applied to bacteria and viruses because their surfaces can be modified by metabolic glycoengineering^29–31^. Therefore, the findings of this study provide a promising route to the generation of living cell-based next-generation innovative materials, technologies, and medicines.

## Methods

### Materials

D-Mannosamine hydrochloride, 2-azidoacetic acid, acetic anhydride, pyridine, alginic acid sodium salt (*M*_w_: 100,000), and azide fluor 545 were purchased from Sigma-Aldrich. 4-(4,6-Dimethoxy-1,3,5-triazin-2-yl)-4-methylmorpholinium chloride n-hydrate (DMT-MM) was purchased from Wako Pure Chemical. WST-1 was purchased from Dojindo. DBCO-carboxyrhodamine 110 and dibenzylcyclooctyne-PEG_4_-amine (DBCO-PEG_4_-amine) were purchased from Click Chemistry Tools. 4-arm PEG-NH_2_ (*M*_w_: 20,000, SUNBRIGHT PTE-200PA) was purchased from NOF Corporation. Other reagents and solvents available in extra-pure grade were obtained commercially and used without further purification.

### Synthesis of Ac_4_ManNAz

D-Mannosamine hydrochloride (500 mg, 2.3 mmol) was added to an aqueous solution of azidoacetic acid (200 µL, 2.4 mmol). DMT-MM (664 mg, 2.4 mmol) was added to the solution, and the reaction mixture was stirred at 45 °C for 2 days. The solution was removed by evaporation to give solid state crude reaction mixtures, and then the objective *N*-azidoacetyl D-mannosamine (ManNAz) was extracted from the crude mixtures by methanol wash three times. Moreover, silica gel chromatography (eluting solution: methanol/chloroform = 2/1, v/v) was performed to obtain pure ManNAz. Acetic anhydride (380 µL, 4.0 mmol) was added to a solution of ManNAz (170 mg, 0.66 mmol) in anhydrous pyridine (5 mL, 62 mmol), and the reaction mixture was stirred overnight at room temperature under nitrogen atmosphere. The solution was concentrated, resuspended in dichloromethane, and washed with 1 M hydrogen chloride, saturated sodium hydrogen carbonate, and then saturated sodium chloride. The organic phase was dried using magnesium sulfate, filtered, and evaporated to give solid state of the objective tetraacetylated *N*-azidoacetyl mannosamine (Ac_4_ManNAz). ^1^H-NMR (500 MHz, CDCl_3_): 1.98−2.22 (−OCOC*H*_3_, 12 H), 3.81 (C2HN*H*CO−, 1 H), 3.92 (−COC*H*_2_N_3_, 2 H), 4.05−4.13 (C3*H*, C4*H*, and C5HC*H*_2_, 4H), 4.27 (C5*H*, 1 H), 4.62 (C2*H*, 1 H), 5.18 (C1*H*, 1H). ESI-MS, calc. 430.4; found 429.08.

### Cell culture

MCF-7 cells (human breast adenocarcinoma cell line), C2C12 cells (mouse myoblast cell line), and HL-60 cells (human promyelocytic leukemia cells) were purchased from ATCC. MCF-7 and C2C12 cells were cultured in DMEM supplemented with 10% FBS and antibiotic solution containing penicillin (100 units mL^−1^) and streptomycin (100 µg mL^−1^) and 2.0 mM L-glutamine at 37 °C in a humidified atmosphere containing 5.0% CO_2_. HL-60 cells were cultured in RPMI 1640 medium supplemented with 10% FBS and antibiotic solution containing penicillin (100 units mL^−1^) and streptomycin (100 µg mL^−1^) and 2.0 mM L-glutamine at 37 °C in a humidified atmosphere containing 5.0% CO_2_.

### Metabolic activity

C2C12 cells (1.0 × 10^4^ cells per well) were seeded on 96-well plate and then 200 μL of DMEM containing Ac_4_ManNAz with varied concentrations was added to the well and cultured for 24 h at 37 °C in a humidified atmosphere containing 5.0% CO_2_. Ten µL of WST-1 solution (WST-1/1-methoxy PMS = 9/1, v/v) was added to the well, and incubated at 37 °C for 2 h. Absorbance at 450 nm and 620 nm was measured by microplate reader (Multiskan FC, Thermo Scientific). Values are average of three separate experiments and are expressed as mean ± SD.

### Preparation of azide-modified cells

C2C12 cells (1.0 × 10^5^) were seeded on 3 cm glass bottom dish, and then 2 mL of DMEM containing Ac_4_ManNAz (0, 10, 20, 30, 50, and 100 μM) was added to the dish and incubated at 37 °C for 3 days. The supernatant was removed and then 2 mL of DMEM was freshly added to the dish. DBCO-carboxyrhodamine 110 (final concentration: 5 µM) was added to the dish and incubated at 37 °C for 1 h. Cells attached on the dish were washed twice with phosphate buffered saline (PBS) and 1 mL of Live Cell Imaging Solution (Life Technologies) was added, and then the cells were observed by confocal laser scattering microscopy (CLSM, ZEISS LSM700). Fluorescence intensity per cell is analyzed by line profiles across a cell in the z-stack images. To prevent saturation of the intensity, we firstly set gain to adjust the detector signal using C2C12 cells with 100 μM Ac_4_ManNAz treatment providing maximum fluorescence intensity. After that, CLSM observation of C2C12 cells with different concentrations of Ac_4_ManNAz treatments was performed using the same gain. The fluorescence intensity is represented as the average of ten cells analyzed and are expressed as mean ± SD.

### Metabolism of azide groups on cell surfaces

C2C12 cells (1.0 × 10^4^) were seeded on 3 cm glass bottom dish, and then 2 mL of DMEM containing Ac_4_ManNAz (100 μM) was added to the dish and incubated at 37 °C for 3 days. The supernatant was removed and washed with PBS twice, and then cells were cultured for 10 days with 2 mL of DMEM without Ac_4_ManNAz. After predetermined times, DBCO-carboxyrhodamine 110 (final concentration: 5 µM) was added to the dish and incubated at 37 °C for 1 h. Cells attached on the dish were washed twice with phosphate buffered saline (PBS) and 1 mL of Live Cell Imaging Solution (Life Technologies) was added, and then the cells were observed by confocal laser scattering microscopy. Fluorescence intensity of the whole cell surfaces are represented as the average of ten cells analyzed using z-stack images and are expressed as mean ± SD.

### Proliferation of azide-modified cells

C2C12 cells were seeded on 10 cm dish, then 10 mL of DMEM with or without Ac_4_ManNAz (100 µM) was added to the dish and incubated at 37 °C for 3 days. Cells were collected by usual trypsin treatment and centrifuged (1000 rpm, 3 min), then supernatant was removed. The pellet of azide-modified cells was re-suspended with DMEM, and seeded on 6 well plate (5 × 10^4^ cells) and cultured at 37 °C in a humidified atmosphere containing 5.0% CO_2_. After predetermined times, cell number was counted by trypan blue assay. Values are average of 3 separate experiments and are expressed as mean ± SD.

### Synthesis of branched alginic acid

4-arm PEG-NH_2_ (2 mg, 0.1 μmol) and DMT-MM (33.2 μg, 0.12 μmol) dissolved in 2 mL of pure water was added to 18 mL of alginic acid (100 mg, 1 μmol) solution, and stirred at room temperature for 6 h. The reaction mixture was dialyzed (MWCO: 14,000) against pure water for 2 days, then the resultant solution was freeze-dried to give white powder of branched alginic acid (bAlg). Molecular structure of bAlg was determined by ^1^H-NMR analysis (D_2_O, 85 °C).

### Synthesis of alkyne-modified branched alginic acid

DBCO-PEG_4_-amine (6.7 mg, 12.7 μmol) and DMT-MM (4.2 mg, 15.3 μmol) dissolved in 1 mL of pure water was added to 19 mL of bAlg (100 mg, 0.15 μmol) solution, and stirred at room temperature for 24 h. The reaction mixture was dialyzed (MWCO: 14,000) against pure water for 2 days, then the resultant solution was freeze-dried to give white powder of DBCO-modified branched alginic acid (bAlg-DBCO). Molecular structure of bAlg-DBCO was determined by ^1^H-NMR analysis (D_2_O, 85 °C). The hydrodynamic diameters of bAlg and bAlg-DBCO solutions (0.05%) in PBS were measured by dynamic light scattering (DLS, ZETASIZER NanoSeries ZEN-3600, Malvern).

### Bioorthogonal click reaction between azide-modified cells and bAlg-DBCO

Azide-modified C2C12 cells (4.5 × 10^4^) were washed twice with PBS, the centrifuged. The pellet was suspended with 500 µL of FITC-labeled bAlg-DBCO (0.5%) in DMEM, incubated at 37 °C for 30 min for bioorthogonal click reaction. After that, cells were centrifuged and washed with PBS twice, then cells were re-suspended with 1 mL of Live Cell Imaging Solution and observed by CLSM.

### In vitro preparation of cell cross-linked hydrogels

Pellet of azide-modified C2C12 cells (0.5 × 10^6^, 1.0 × 10^6^ or 2.0 × 10^6^) was suspended with 100 µL of bAlg-DBCO solution (1% and 2%, w/v) in HEPES buffer (200 mM, pH 7.4) and cells were completely dispersed by gentle pipetting. The cell dispersions were added into test tube and incubated at 37 °C. Gel formation was checked by usual test tube inverting methods. Five hundred mL of DMEM was carefully added on the hydrogels prepared in test tube, then hydrogels was taken out from the test tube by vortex shaking, placed on the slide glass and taken a picture. The same procedure was carried out to prepare cell cross-linked hydrogels using MCF-7 and HL-60. As controls, normal C2C12 cells (0.5 × 10^6^, 1.0 × 10^6^ or 2.0 × 10^6^) was suspended with 100 µL of bAlg-DBCO solution (1% and 2%, w/v) in HEPES buffer (200 mM, pH 7.4), and test tube inverting methods were performed. Moreover, azide-modified C2C12 cells (2.0 × 10^6^) was also suspended with 100 µL of bAlg solution (2%, w/v) in HEPES buffer (200 mM, pH 7.4), and test tube inverting methods were performed as controls.

### Rheological characterization of cell cross-linked hydrogels

Pellet of azide-modified C2C12 cells (0.5 × 10^6^, 1.0 × 10^6^ or 2.0 × 10^6^) was suspended with 100 µL of bAlg-DBCO solution (1% and 2%, w/v) in HEPES buffer (200 mM, pH 7.4) and cells were completely dispersed by gentle pipetting. Rheological test of the cell dispersions were performed on MCR 302 rheometer (Anton Paar) using a standard steel parallel-plate geometry of 25 mm in diameter. Oscillatory time and frequency were performed at 37 °C, and the storage modulus (*G*′) and loss modulus (*G*″) were recorded. The cell dispersions were cast between the lower plate and upper plate. To prevent evaporation of water and better temperature control during testing, the plates were enclosed in a chamber. Time zero was taken as the moment at which the cell dispersions were cast on the plate. The time sweep data collection was started from time zero to 3600 s to monitor the gelation process. The strain was maintained at 5% and operated at 10 rad/s. Frequency sweep was performed using the same hydrogels 2 h after the addition of bAlg-DBCO solution to pellet of azide-modified C2C12 cells to determine the stability of the hydrogels. The strain was maintained at 5%, and the frequency was swept from 100 to 0.1 rad/s. As controls, azide-modified C2C12 cells (2.0 × 10^6^) was also suspended with 100 µL of bAlg solution (2%, w/v) in HEPES buffer (200 mM, pH 7.4), and rheological characterization was performed. Time-dependent changes in the *G*′ and *G*″ values of the cell cross-linked hydrogels cultured in DMEM with or without Ac_4_ManNAz (100 μM) were examined for 7 days. Frequency sweep was performed with the fixed strain at 5%, and the frequency was swept from 100 to 0.1 rad/s.

### Preparation of cell cross-linked hydrogels using freezing-thawing azide-modified cells

C2C12 cells were seeded on 10 cm dish, and then DMEM containing 100 µM of Ac_4_ManNAz was added to the dish and incubated at 37 °C for 3 days. Cells were collected by usual trypsin treatment and centrifuged (1000 rpm, 3 min), then supernatant was removed. The pellet of azide-modified cells was suspended with CELLBANKER (Wako Pure Chemical) and added to cryotube. The tube was gradually cooled in BICELL (NIHON FREEZER), then stored −80 °C. After that, azide-modified cells were thawed and the cells (2.0 × 10^6^) was suspended with 100 µL of bAlg-DBCO solution (2%, w/v) in HEPES buffer (200 mM, pH 7.4), then test tube inverting methods were performed.

### Cell viability in cell cross-linked hydrogels

NPellet of azide-modified C2C12 cells (2.0 × 10^6^) was suspended with 100 µL of bAlg-DBCO solution (2%, w/v) in HEPES buffer and cells were completely dispersed by gentle pipetting. The cell dispersion solution was put on glass bottom dish and incubated at 37 °C for 1 h. Two mL of DMEM was added on the gels and cultured at 37 °C in a humidified atmosphere containing 5.0% CO_2_. After a sufficient time, Calcein-AM (DOJINDO) and propidium iodide (PI, DOJINDO) were added in the medium and reacted for 30 min at 37 °C, then gels were washed with PBS twice and CLSM observation was carried out in 1 mL of Live Cell Imaging Solution. Values are average of three separate experiments and are expressed as mean ± SD. To prepare C2C12 cells-encapsulating control physical gels, azide-modified C2C12 cells (2.0 × 10^6^) were well suspended with 100 µL of bAlg solution (2%, w/v) in HEPES buffer in the presence of CaCl_2_ solution (0.5%). The cell viability of C2C12 cells physically encapsulated in the control gels was also examined with the same live/dead assay.

### Cell proliferation in cell cross-linked hydrogels

Pellet of azide-modified C2C12 cells (2.0 × 10^5^) was suspended with 10 µL of bAlg-DBCO solution (2%, w/v) in HEPES buffer and cells were completely dispersed by gentle pipetting. The cell dispersions were poured into 96-well plate and incubated at 37 °C for 1 h. Two hundred μL of DMEM was added on the gels and cultured for 7 days at 37 °C in a humidified atmosphere containing 5.0% CO_2_. The supernatant was carefully changed with fresh DMEM with or without Ac_4_ManNAz (100 μM) every day. After predetermined times, the gels were carefully transferred into a new well, then 100 μL of fresh DMEM and 5 µL of WST-1 solution was added to the well and incubated at 37 °C for 2 h. The gels were demolished thoroughly by vigorous pipetting, then the 96-well plate was centrifuged and the supernatant (100 μL) was carefully sucked and poured into new 96-well plate. Absorbance at 450 nm and 620 nm was measured by microplate reader. Values are average of three separate experiments and are expressed as mean ± SD. To prepare C2C12 cells-encapsulating control physical gels, azide-modified C2C12 cells (2.0 × 10^5^) were well suspended with 10 µL of bAlg solution (2%, w/v) in HEPES buffer in the presence of CaCl_2_ solution (0.5%). The cell proliferation of C2C12 cells physically encapsulated in the control gels was also examined with the same WST-1 assay.

### Weight change in cell cross-linked hydrogels

Pellet of azide-modified C2C12 cells (1.0 × 10^6^) was suspended with 50 µL of bAlg-DBCO solution (2%, w/v) in HEPES buffer and cells were completely dispersed by gentle pipetting. The cell dispersion solution was poured into plastic microtube and incubated at 37 °C for 1 h. In total 1 mL of DMEM was added on the gels and cultured for 7 days at 37 °C in a humidified atmosphere containing 5.0% CO_2_. DMEM was changed with fresh one every day. After predetermined time, the remained gels were carefully transferred into a new plastic microtube, and washed with PBS twice, then the weight of swollen gels was measured. Moreover, gels were lyophilized and the weight of the dry gel was also measured. Values are average of three separate experiments and are expressed as mean ± SD. To prepare C2C12 cells-encapsulating control physical gels, azide-modified C2C12 cells (1.0 × 10^6^) were well suspended with 50 µL of bAlg solution (2%, w/v) in HEPES buffer in the presence of CaCl_2_ solution (0.5%). One mL of DMEM was added on the gels and cultured for 7 days at 37 °C in a humidified atmosphere containing 5.0% CO_2_. The changes in the weight of swollen and the dry gels were examined with the same procedures. Moreover, bAlg physical gels (2%, w/v) without cells were prepared in the presence of CaCl_2_ solution and the changes in the weight of swollen and the dry gels were examined with the same procedures.

### Cell adhesion in cell cross-linked hydrogels

Pellet of azide-modified C2C12 cells (2.0 × 10^6^) was suspended with 100 µL of bAlg-DBCO solution (2%, w/v) in HEPES buffer and cells were completely dispersed by gentle pipetting. The cell dispersion solution was put onto collagen-type I-coated glass bottom dish (Cosmo Bio) or 2-methacryloyloxyethyl phosphorylcholine (MPC) polymer-coated dish (Thermo Fisher Scientific) and incubated at 37 °C for 1 h. Two mL of DMEM was carefully added on the gels and cultured at 37 °C in a humidified atmosphere containing 5.0% CO_2_. After 24 h, cells in the hydrogels that exist on the interface between gels and the top surface of dish were observed by CLSM. To prepare C2C12 cells-encapsulating control physical gels, azide-modified C2C12 cells (2.0 × 10^6^) were well suspended with 100 µL of bAlg solution (2%, w/v) in HEPES buffer in the presence of CaCl_2_ solution (0.5%). Cell adhesion in the control physical gels on both the collagen- or MPC polymer-coated dishes was also observed by CLSM.

### Gel adhesion

Pellet of azide-modified C2C12 cells (2.0 × 10^6^) was suspended with 100 µL of bAlg-DBCO solution (2%, w/v) in HEPES buffer and cells were completely dispersed by gentle pipetting. The cell dispersions were put onto collagen-type I-coated glass bottom dish and incubated at 37 °C for 1 h. Two mL of DMEM was added on the gels and cultured at 37 °C in a humidified atmosphere containing 5.0% CO_2_. After 24 h, adhesion of the gels onto the surface of dish was examined by dish inverting method. After that, 1 mL of trypsin solution was added to dish and incubated at 37 °C for 10 min, then adhesion of the gel onto surface of dish was examined. The harvested gel was gently washed with DMEM twice and put onto new collagen-coated dish, then cultured at 37 °C in a humidified atmosphere containing 5.0% CO_2_. After 24 h, adhesion of the gel onto surface of dish was examined. To prepare C2C12 cells-encapsulating control physical gels, azide-modified C2C12 cells (2.0 × 10^6^) were well suspended with 100 µL of bAlg solution (2%, w/v) in HEPES buffer in the presence of CaCl_2_ solution (0.5%). Moreover, bAlg physical gels (2%, w/v) without cells were also prepared in the presence of CaCl_2_ solution as control gels. After 24 h, adhesion of the control gels onto the surface of dish was examined by dish inverting method.

### In vivo preparation of cell cross-linked hydrogels

All animal experiments and all experimental protocols were approved by Konan University, and conformed to the Guidelines for the Care and Use of Laboratory Animals published by the National Institutes of Health. Female nude mice (ICR-nu/nu) at 4 weeks of age were purchased from Charles River. To generate retroviruses encoding Lifeact-GFP for the visualization of F-actin structures, the nucleotide sequence 5′-ATGGGTGTCGCAGATTTGATCAAGAAATTCGAAAGCATCTCAAAGGAAGAA-3′, which encodes the Lifeact peptide, was subcloned into the pBabe blast vector containing a C-terminal GFP tag. pBabe Lifeact-GFP blast was cotransfected with psi-2 helper plasmid into human kidney 293 T cells (ATCC) using the HilyMax transfection reagent (Dojindo, Kumamoto, Japan). Forty-eight hours after transfection, the supernatant was collected and then used to infect C2C12 cells in the presence of 8 μg mL^−1^ polybrene (Sigma). Infected C2C12 cells were selected using 3 μg mL^−1^ blasticidin (Invitrogen, Carlsbad, CA) for 3 days. Nude mice (ICR-nu/nu, 6 weeks old, female) were anesthetized with isoflurane. Azide-modified C2C12 cells constitutively expressing Lifeact-GFP (4.0 × 10^6^ cells) were suspended with 200 μL of bAlg-DBCO solution and immediately injected subcutaneously into the muscle layer of the back of each mouse. After a predetermined time, the mice were killed, the hydrogels were excised, and the hydrogels were analyzed.

### Transplantation of cell cross-linked hydrogels in a mouse model of skeletal muscle injury

Nude mice (BALB/c-nu/nu, 5 weeks old, female) were anesthetized with isoflurane. The skin of the hind limb was opened, and the femoral muscle was gently exposed. In host femoral muscle, a local injury (ca. 7 mm long, 4 mm wide, and 5 mm deep) was created by removing skeletal muscle fibers. The skin was then carefully closed and the incision was sutured (4/0 silk). One day after the surgery, 250 μL of CxGels precursor solution, Lifeact-GFP-expressing N_3_-C2C12 cells (5.0 × 10^6^) suspended with bAlg-DBCO solution, was injected into the injury site using a syringe with a 26-gauge needle. As control gels, physically cross-linked bAlg gels encapsulating Lifeact-GFP-expressing N_3_(+)C2C12 cells (5.0 × 10^6^) were prepared in the presence of CaCl_2_ solution (0.5%), then the gels were transplanted into the injury site. After transplantation, the muscle strength of the injured hind limbs was quantitatively measured every day using a grip-strength meter (MELQUEST, GMP-100B). Values are average of three separate experiments in triplicate and are expressed as mean ± SD. At the end of the experiment (after 15 days), the injury site was carefully opened and the regenerated skeletal muscle tissues were analyzed by CLSM observation.

### Preparation of tissue cross-linked hydrogels

Female mice (ICR) at 5 weeks of age were purchased from Charles River. In total 6 mg of AC_4_ManNAz was dissolved in PBS/DMSO mixture (42/58, v/v). Mice were anesthetized using isoflurane, then 200 μL of AC_4_ManNAz solution was administrated by intraperitoneal injection. This administration was carried out for continuous 7 days. The mice were killed and heart, lung, kidney, and femoral muscle were excised carefully. These tissues were shredded and suspended with 500 µL of FITC-labeled bAlg-DBCO (0.1%) in DMEM, incubated at 37 °C for 30 min for biorthogonal click reaction. After that, tissues were centrifuged and washed with PBS twice, then tissues were re-suspended with 1 mL of Live Cell Imaging Solution and observed by CLSM. For preparation of tissue cross-linked hydrogels, these tissues were shredded and washed with PBS twice. Pellet of azide-modified tissue was suspended with 100 µL of bAlg-DBCO solution (2%, w/v) in HEPES buffer and completely dispersed by gentle pipetting. The tissue dispersion solution was added into test tube and incubated at 37 °C for 1 h. Gel formation was checked by usual test tube inverting methods.

### Data availability

The authors declare that the data supporting the findings of this study are available within the paper and its supplementary information files.

## Electronic supplementary material

Supplementary Information

Description of Additional Supplementary Files

Supplementary Movie 1

Supplementary Movie 2

Supplementary Movie 3

Supplementary Movie 4

Supplementary Movie 5

Supplementary Movie 6
