# Supplementary material for: Living functional hydrogels generated by bioorthogonal cross-linking reactions of azide-modified cells with alkyne-modified polymers
Source: Nat Commun. 2018 Jun 6;9:2195. doi: 10.1038/s41467-018-04699-3 (PMC5989231; doi:10.1038/s41467-018-04699-3)
Supplement: Supplementary file 1 — Supplementary Information [file 41467_2018_4699_MOESM1_ESM.pdf]

Living functional hydrogels generated by bioorthogonal cross-linking reactions of azide-modified cells with alkyne-modified polymers

Koji Nagahama*, Yuuka Kimura & Ayaka Takemoto

Department of Nanobiochemistry, Frontiers of Innovative Research in Science and Technology (FIRST), Konan University, 7-1-20 Minatojima-Minamimachi, Chuo-ku, Kobe 650-0047, Japan. E-mail: nagahama@center.konan-u.ac.jp.

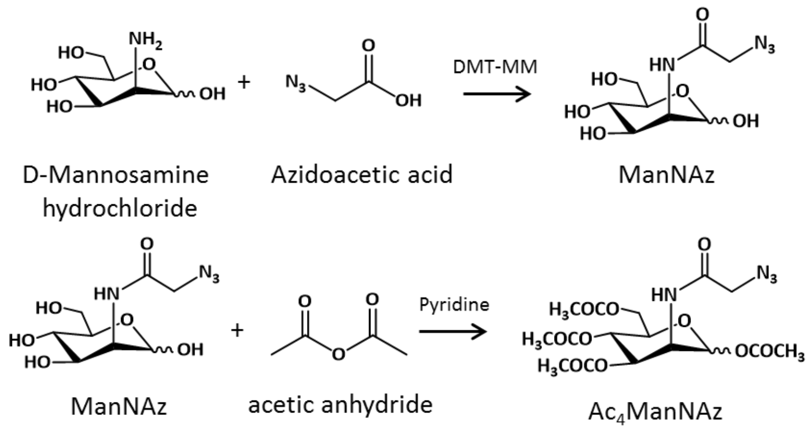

Supplementary Figure 1. Synthesis of AC_4ManNAz.

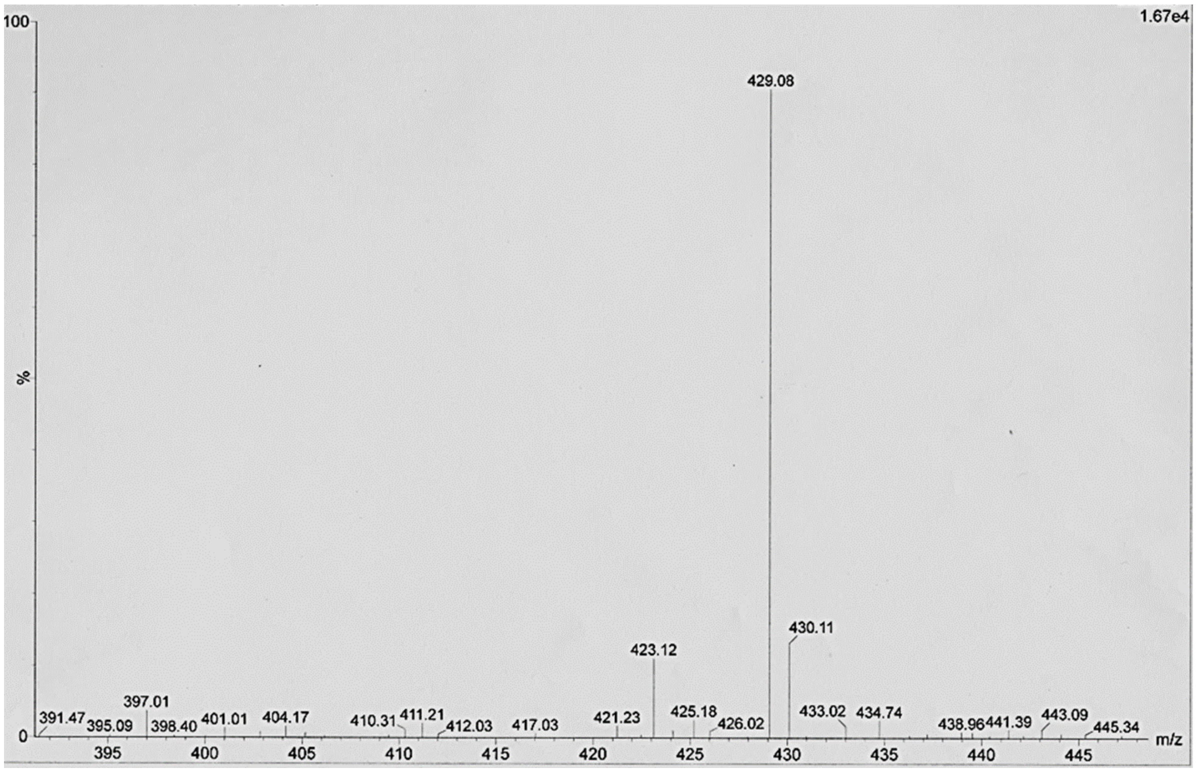

Supplementary Figure 2. ESI-MS spectrum of Ac_4ManNAz.

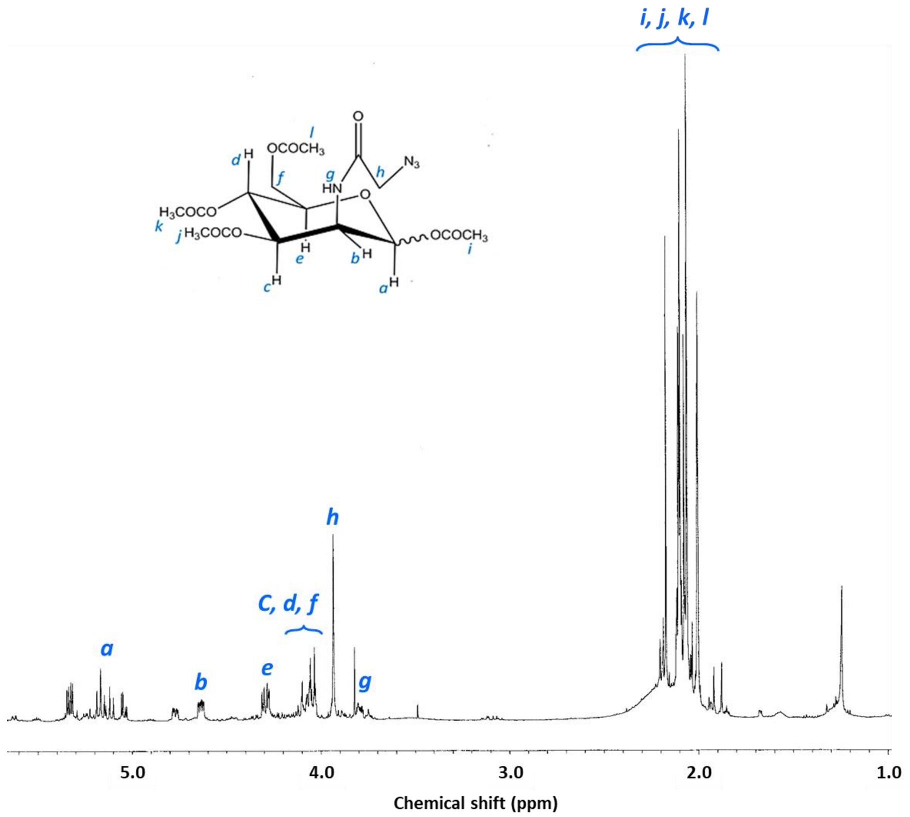

Supplementary Figure 3. ^{1}H-NMR spectrum of Ac_4ManNAz measured in $CDCl_3$.

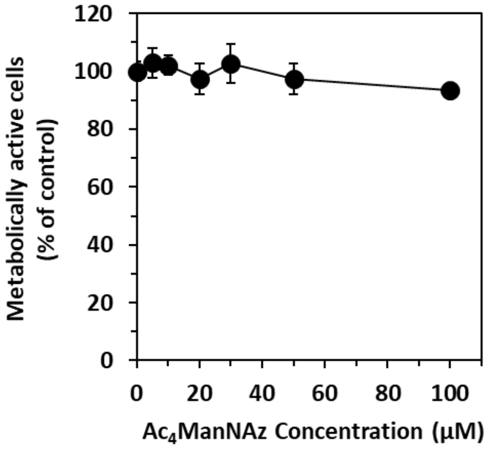

Supplementary Figure 4. Metabolic activity of C2C12 cells at 3 days after treatments with Ac_4ManNAz with varied concentrations analyzed by WST-1 assay. Error bars: standard deviation (n = 3).

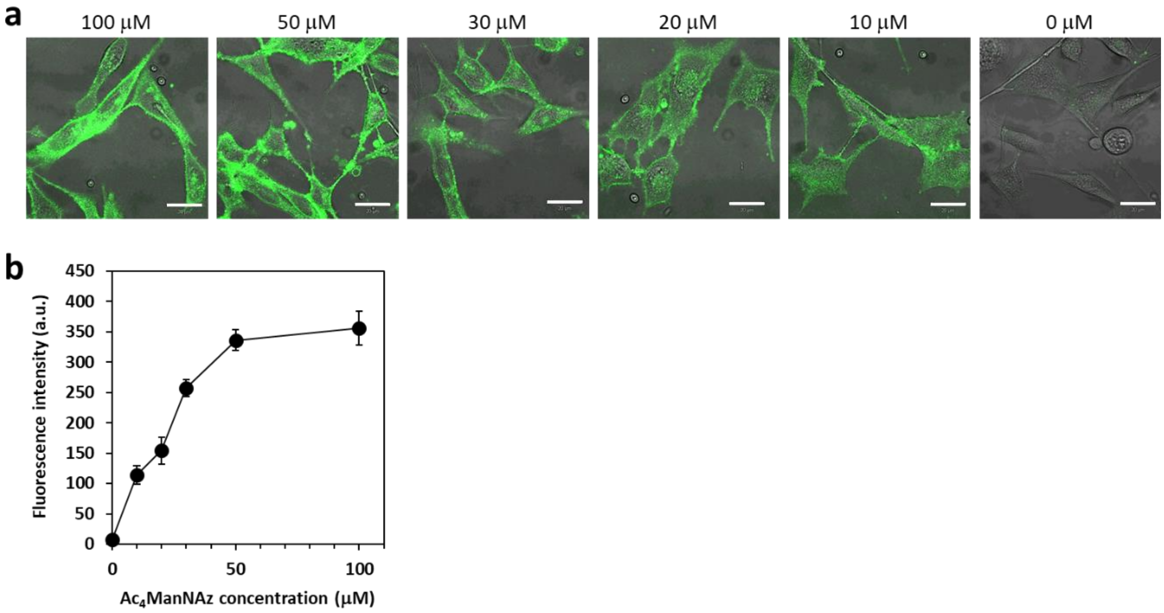

Supplementary Figure 5. (a) CLSM images of C2C12 cells treated with different concentration of $Ac_4ManNAz$ for 3 days and followed by reacted with DBCO-PEG_4-Carboxyrhodamine 110 for 1 h. Scale bars indicate 20 μm. (b) Relative fluorescence intensity of C2C12 cells derived from carboxyrhodamine linked to the cell surface. Error bars: standard deviation (*n* = 3).

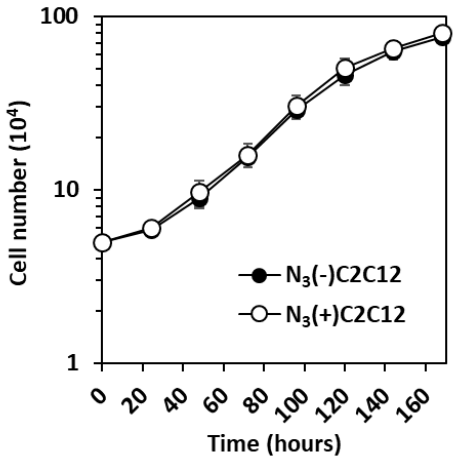

Supplementary Figure 6. Growth curves of normal C2C12 cells [N_3(-)C2C12] and azide-modified C2C12 cells [N_3(+)C2C12]. N_3(+)C2C12 cells were prepared by treatment with 100 μM of $Ac_4ManNAz$ for 3 days. Error bars: standard deviation (*n* = 3).

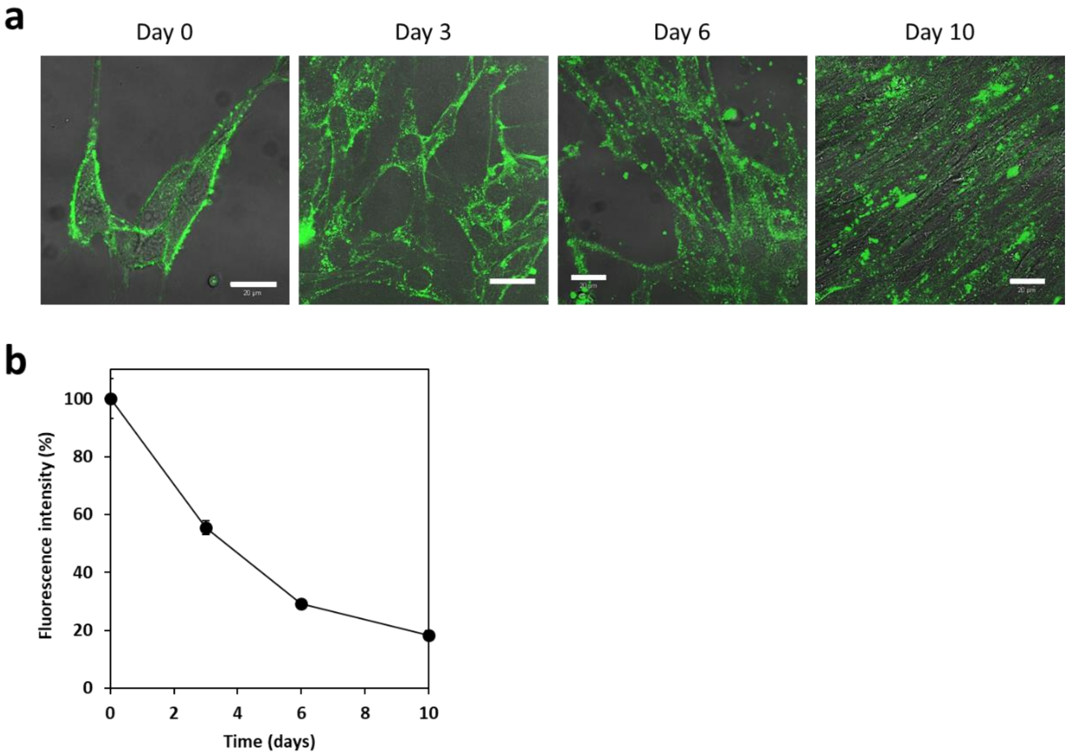

Supplementary Figure 7. (a) CLSM images of C2C12 cells treated with $Ac_4ManNAz$ (100 μM) for 3 days followed by cultured in normal DMEM (absence of $Ac_4ManNAz$) for 10 days. At the predetermined time, C2C12 cells were reacted with DBCO-PEG_4-Carboxyrhodamine 110 for 1 h. Scale bars indicate 20 μm. (b) Changes in relative fluorescence intensity of the C2C12 cells derived from carboxyrhodamine linked to the cell surface. Error bars: standard deviation (n = 3).

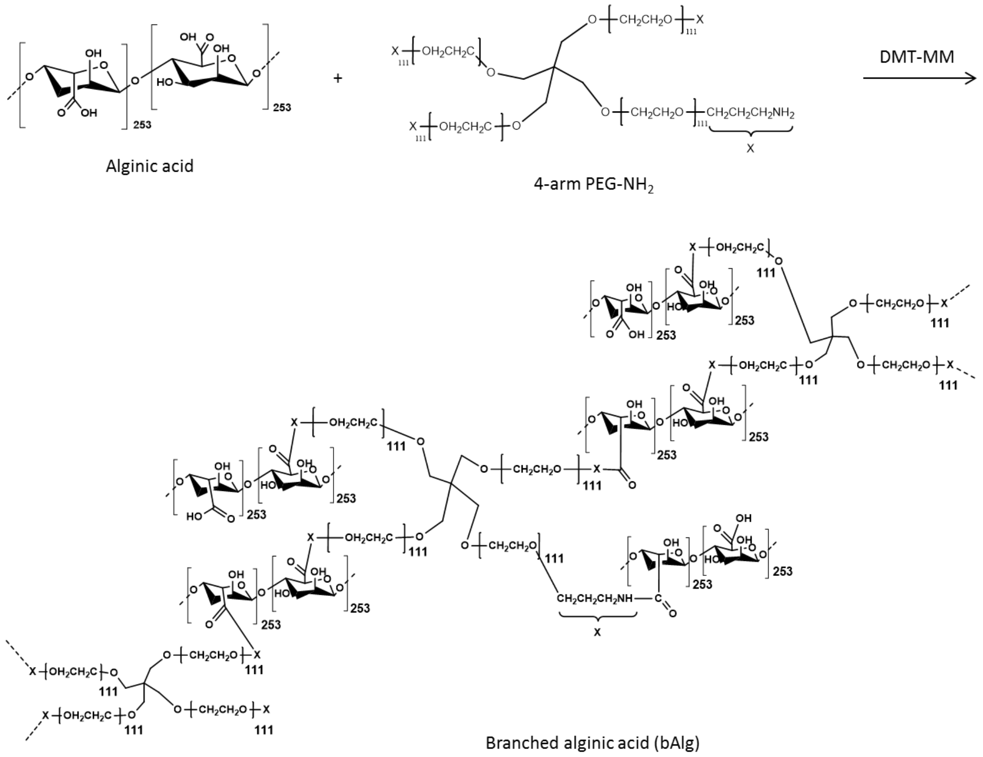

Supplementary Figure 8. Synthesis of branched alginic acid (bAlg).

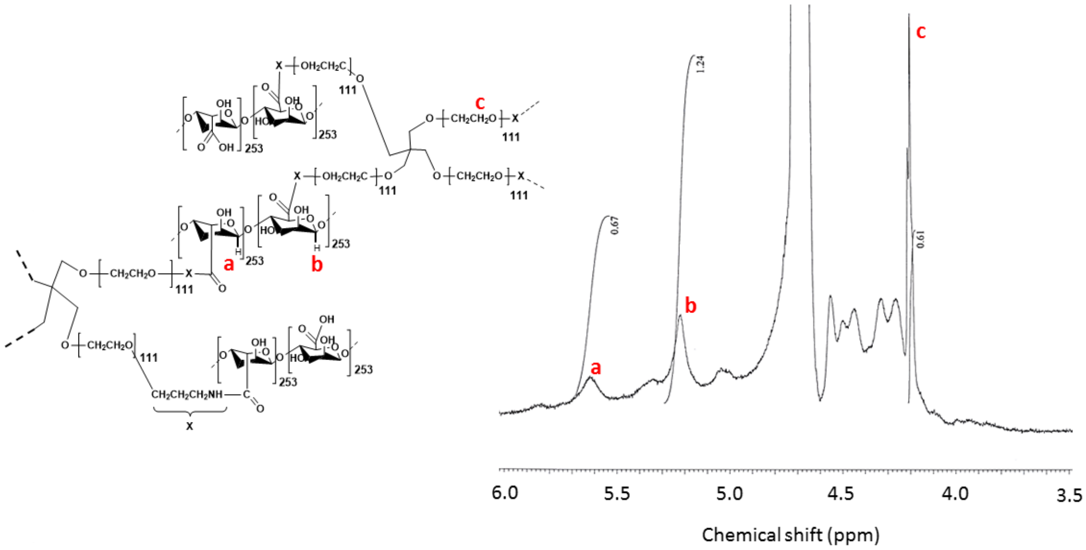

Supplementary Figure 9. ^{1}H-NMR spectrum of branched alginic acid (bAlg) measured in D_2O.

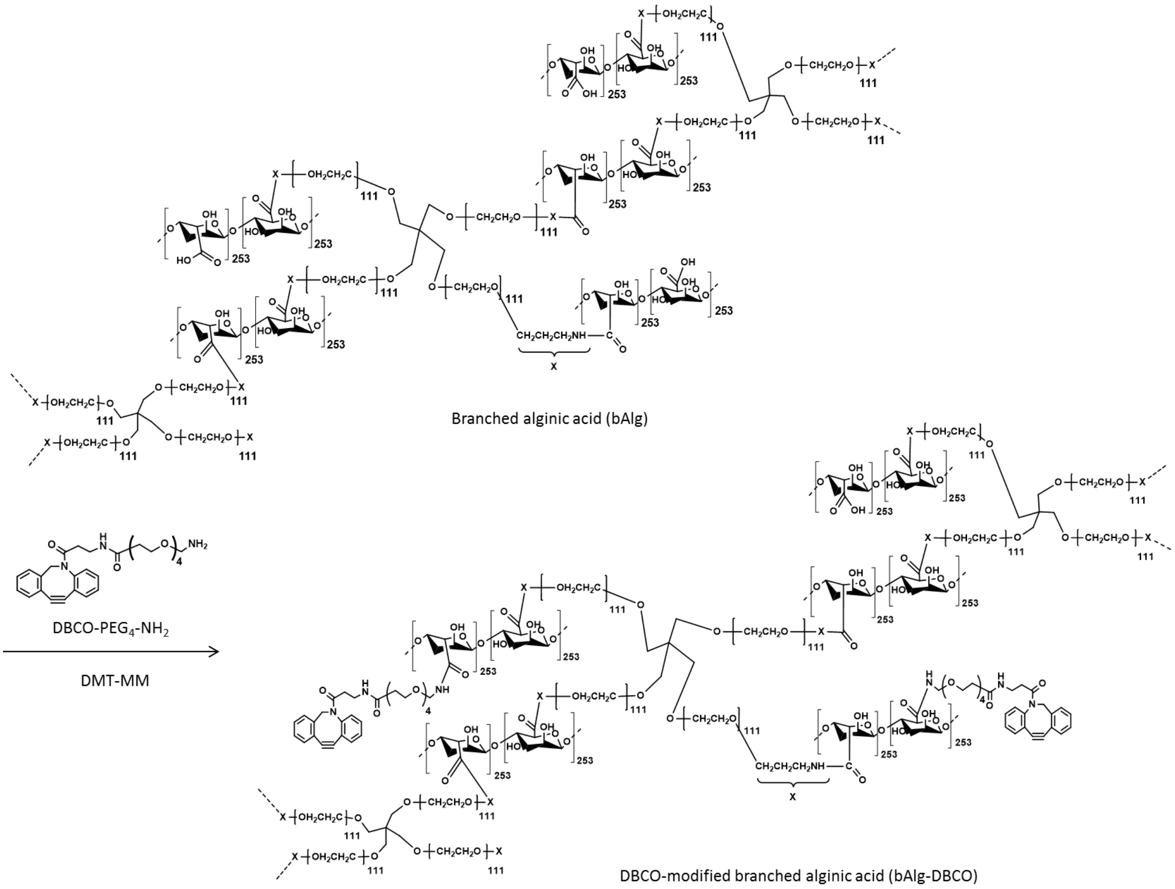

Supplementary Figure 10. Synthesis of DBCO-modified branched alginic acid (bAlg-DBCO).

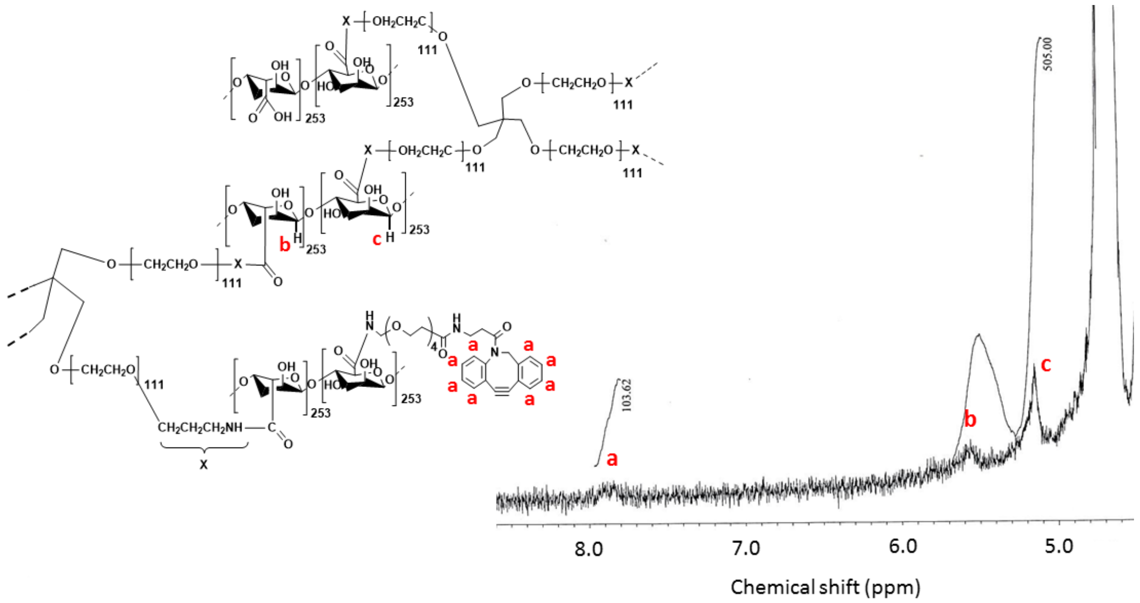

Supplementary Figure 11. ^{1}H-NMR spectrum of bAlg-DBCO measured in D_2O.

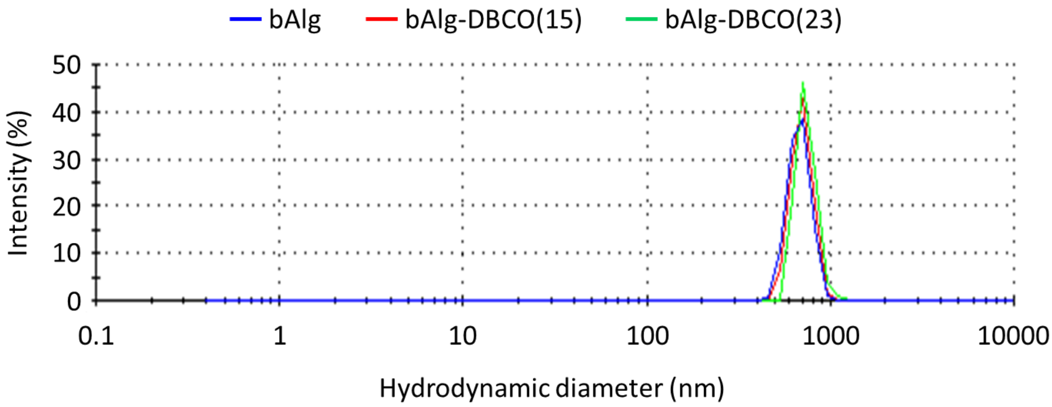

Supplementary Figure 12. Hydrodynamic diameter of bAlg and bAlg-DBCOs with different DBCO numbers introduced to a bAlg molecule in PBS (0.05%) and their distribution analyzed by DLS.

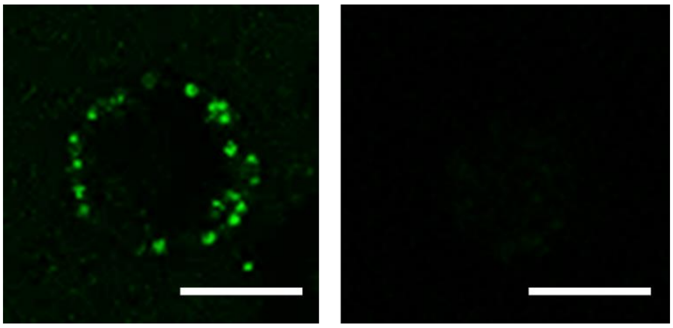

Supplementary Figure 13. CLSM images of (left) azide-modified C2C12 cells treated with bAlg-DBCO-FITC, and (right) azide-modified C2C12 cells treated with bAlg-FITC. Scale bars indicate 10 μm.

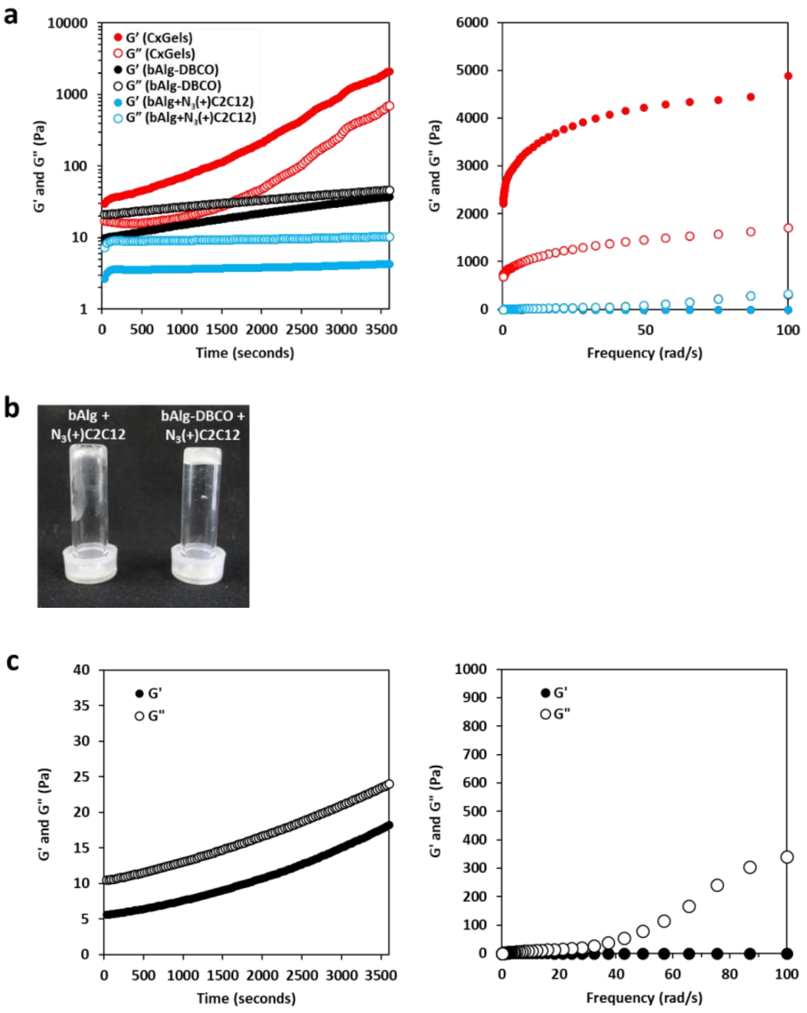

Supplementary Figure 14. Rheological characterization of the CxGels. (a) (left) Gelation kinetics determined through oscillatory time sweep of CxGels [bAlg-DBCO (2%) + N_3(+)C2C12 (2.0 × 10^6)], bAlg-DBCO solutions, and controls [bAlg (2%) + N_3(+)C2C12 (2.0 × 10^6)] at 37 ºC under constant strain (5%) and frequency (10 rad/s). The crossover time point of the storage modulus (*G*') and the loss modulus (*G*") curves is defined as the mechanical gel point. (right) Frequency sweep of CxGels [bAlg-DBCO (2%) + N_3(+)C2C12 (2.0 × 10^6)] at 2 h after the start of the cross-link reaction, bAlg-DBCO solutions, and controls [bAlg (2%) + N_3(+)C2C12 (2.0 × 10^6)] showing the shear frequency dependence of the storage modulus (*G*'). (b) Photographs of the CxGels [bAlg-DBCO (2%) + N_3(+)C2C12 (2.0 × 10^6)] and controls [bAlg (2%) + N_3(+)C2C12 (2.0 × 10^6)] prepared in test tube at 37 ºC. (c) (left) oscillatory time sweep and (right) oscillatory frequency sweep of cell dispersions [bAlg-DBCO (2%) + N_3(-)C2C12 (2.0 × 10^6)] at 37 ºC under constant strain (5%)

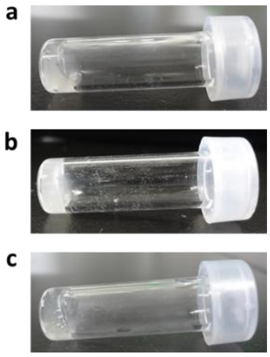

Supplementary Figure 15. (a) Photograph of the CxGels prepared by click reaction between azide-modified MCF-7 cells (2.0×10^6) and bAlg-DBCO (2%). (b) Photograph of the CxGels prepared by click reaction between azide-modified HL-60 cells (2.0×10^6) and bAlg-DBCO (2%). (c) Photograph of the CxGels prepared by click reaction between freezing-thawing azide-modified C2C12 cells (2.0×10^6) and bAlg-DBCO (2%).

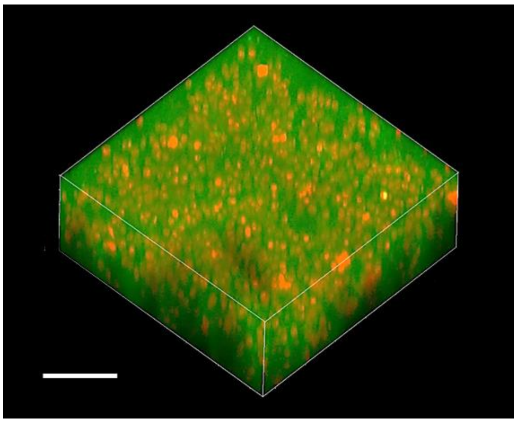

Supplementary Figure 16. CLSM images of the CxGels prepared by click reaction between azide-modified C2C12 cells (2.0×10^6) stained with CytoTell red and bAlg-DBCO-FITC (2%). Green: bAlg-DBCO, red: azide-modifed C2C12 cells. Scale bar indicate 250 μm.

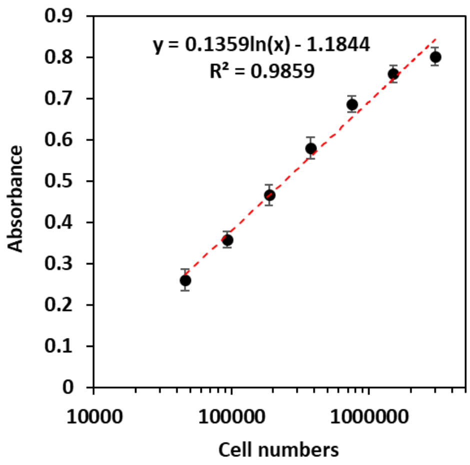

Supplementary Figure 17. Standard curve of WST-1 assay with the same experimental condition as cell proliferation in the CxGels. Error bars: standard deviation ($n = 3$).

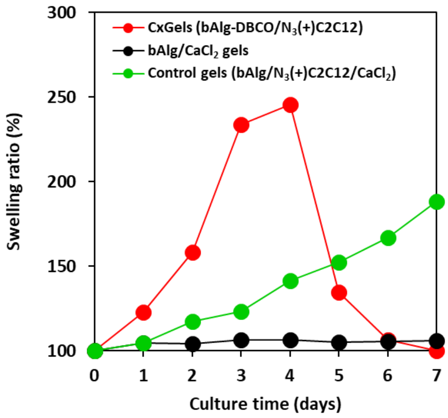

Supplementary Figure 18. Dynamic swelling of the CxGels [bAlg-DBCO (2%) + N_3(+)C2C12 (2.0×10^6)], bAlg (2%)/Ca^{2+} gels, and N_3(+)C2C12 (2.0×10^6)-loaded bAlg (2%)/Ca^{2+} gels in cell culture medium at 37 ºC. Error bars: standard deviation ($n = 3$).

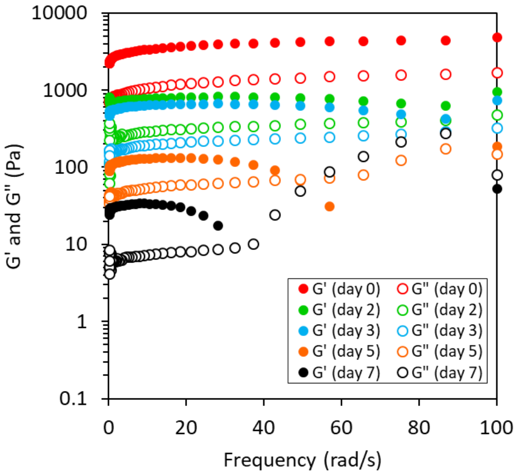

Supplementary Figure 19. Rheological characterization of the CxGels [bAlg-DBCO (2%) + N_3(+)C2C12 (2.0 × 10^6)] cultured in DMEM without further addition of Ac_4ManNAz for 2, 3, 5, and 7 days. Frequency sweep of CxGels showing the shear frequency dependence of the storage modulus (*G*') and the loss modulus (*G*").

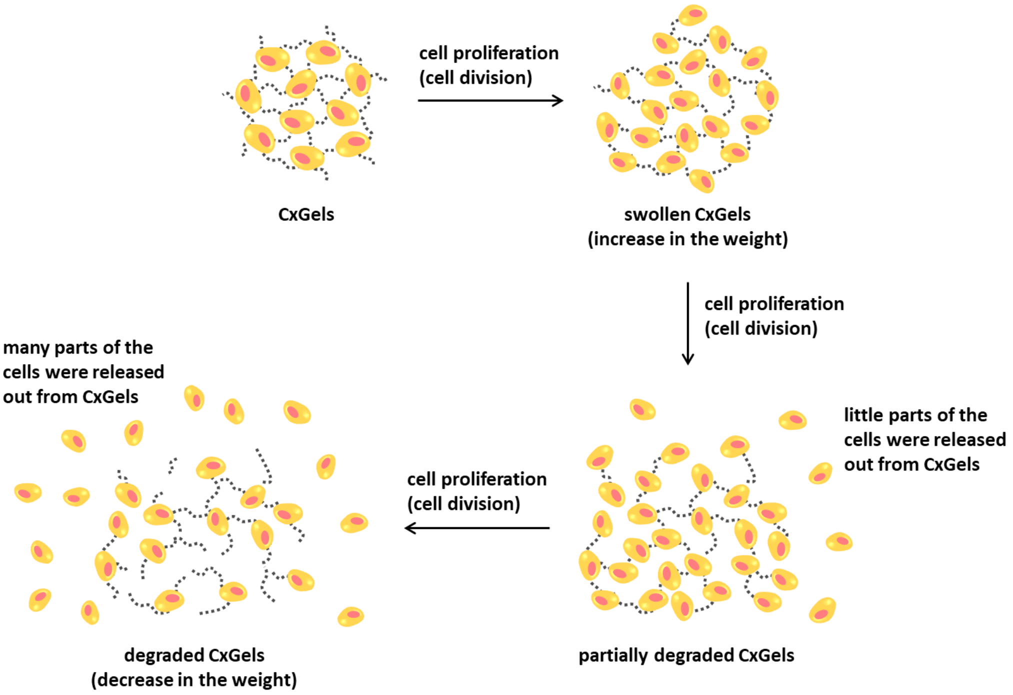

Supplementary Figure 20. Schematic illustration for the swelling and following degradation of CxGels via cell proliferation.

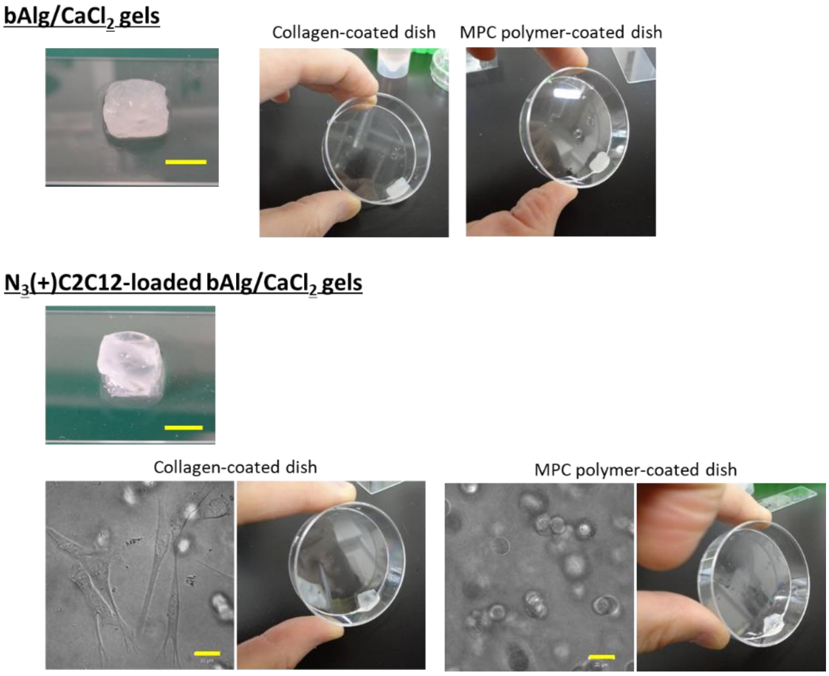

Supplementary Figure 21. Photographs of bAlg (2%)/Ca^{2+} gels and N3(+)C2C12 (2.0 × 10^6)-loaded bAlg (2%)/Ca^{2+} gels placed on cell culture dish with collagen- or MPC polymer coating for 24 h at 37 ºC. Scale bars indicate 5 mm. C2C12 cells in the N3(+)C2C12 (2.0 × 10^6)-loaded bAlg (2%)/Ca^{2+} gels were observed by CLSM analysis. Scale bars indicate 20 μm.

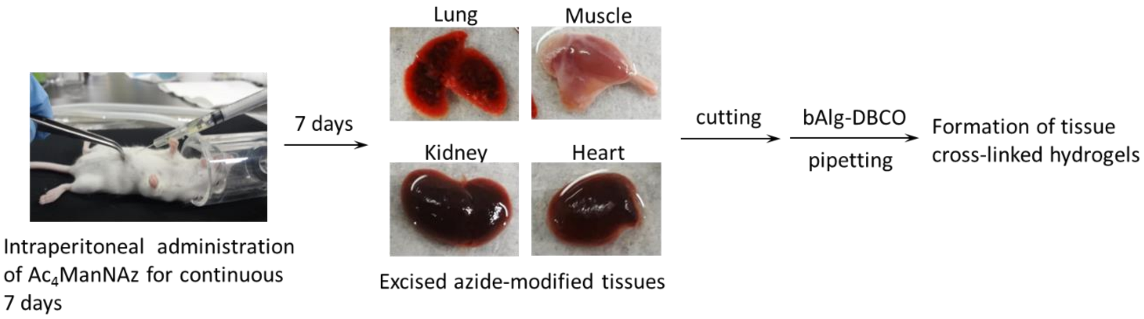

Supplementary Figure 22. Schematic illustration of preparation of tissue cross-linked hydrogels made by click reaction between azide-modified tissue shredded and bAlg-DBCO *ex vivo*.

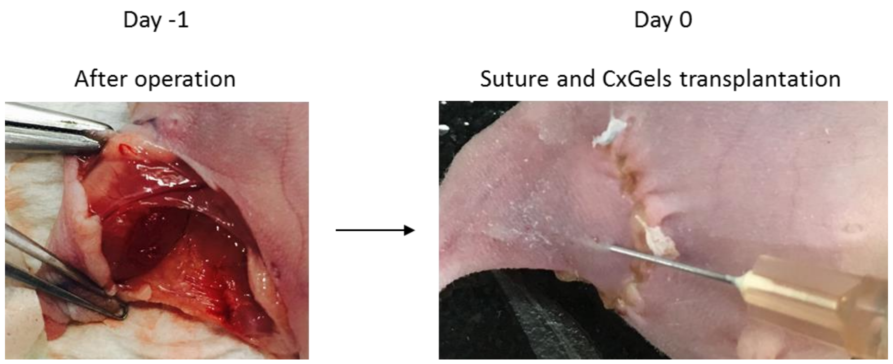

Supplementary Figure 23. Schematic diagram of the experimental procedure, from the establishment of a femoral muscle injured mouse model to transplantation of CxGels.
