# Supplementary material for: Living functional hydrogels generated by bioorthogonal cross-linking reactions of azide-modified cells with alkyne-modified polymers
Source: Nat Commun. 2018 Jun 6;9:2195. doi: 10.1038/s41467-018-04699-3 (PMC5989231; doi:10.1038/s41467-018-04699-3)
Supplement: Supplementary file 2 — Description of Additional Supplementary Files [file 41467_2018_4699_MOESM2_ESM.docx]

**Description of Additional Supplementary Files**

File Name: Supplementary Movie 1

Description: Preparation process of cell cross-linked hydrogels composed of 2.0 × 10^6^ azide-modified C2C12 cells and 2% bAlg-DBCO solutions.

File Name: Supplementary Movie 2

Description: Cell cross-linked hydrogels, prepared through click reaction between 2.0 × 10^6^ azide-modified C2C12 cells and 2% bAlg-DBCO solution, did not adhere on a MPC polymer-coated dish.

File Name: Supplementary Movie 3

Description: Cell cross-linked hydrogels, prepared through click reaction between 2.0 × 10^6^ azide-modified C2C12 cells and 2% bAlg-DBCO solution, maintained adhesion on a collagen-coated dish even after physical stimuli underwater.

File Name: Supplementary Movie 4

Description: Cell cross-linked hydrogels, prepared through click reaction between 2.0 × 10^6^ azide-modified C2C12 cells and 2% bAlg-DBCO solution, maintained adhesion on a collagen-coated dish even after physical stimuli in the air.

File Name: Supplementary Movie 5

Description: Cell cross-linked hydrogels, prepared through click reaction between 2.0 × 10^6^ azide-modified C2C12 cells and 2% bAlg-DBCO solution, detached from a collage-coated dish after trypsin/EDTA treatment.

File Name: Supplementary Movie 6

Description: Trypsin/EDTA treated Cell cross-linked hydrogels, prepared through click reaction between 2.0 × 10^6^ azide-modified C2C12 cells and 2% bAlg-DBCO solution, adhered again on a collagen-coated dish.
